# Microvesicles released from hormone-refractory prostate cancer cells facilitate mouse pre-osteoblast differentiation

**DOI:** 10.1007/s10735-012-9415-1

**Published:** 2012-04-17

**Authors:** Tomohiro Itoh, Yuko Ito, Yoshinori Ohtsuki, Masashi Ando, Yasuyuki Tsukamasa, Nami Yamada, Tomoki Naoe, Yukihiro Akao

**Affiliations:** 1 Faculty of Agriculture, Kinki University, 3327-204 Nakamachi, Nara, 631-8505 Japan; 2 Osaka Medical College, 2-7 Daigaku-cho, Takatsuki, Osaka 596-8686 Japan; 3 United Graduate School of Veterinary Sciences, Gifu University, Gifu, Japan; 4 Department of Hematology and Oncology, Graduate School of Medicine, Nagoya University, 65 Tsurumai-cho, Showa-ku, Nagoya 466-8550 Japan; 5 United Graduate School of Drug Discovery and Medical Information Sciences, Gifu University, 1-1 Yanagido, Gifu, 501-1193 Japan

**Keywords:** Prostate cancer, Microvesicles, Osteoblast differentiation, Ets1, Osteoblastic bone metastasis

## Abstract

Bone metastasis is often occurs in patients with prostate cancer. There is a vicious cycle for bone metastases involving prostate cancer cells, osteoblasts, and osteoclasts. Acting among those cells during the process of metastasis are several molecules such as bone morphogenetic proteins, platelet-derived growth factor, endothelin-1, matrix metalloproteases, vascular endothelial growth factor, transforming growth factor-β, and insulin-like growth factors. Cell-derived microvesicles are endogenous carriers transporting proteins, mRNAs and miRNAs between cells, which is a candidate for participation in the bone metastasis of these cells. Here, we demonstrated that prostate cancer cells in vitro released microvesicles into the culture medium (PCa-MVs), which was shown by electron microscopic study and nanoparticle tracking analysis. In this study, we found for the first time that these PCa-MVs enhanced osteoblast differentiation mainly through the delivery of PCa cell-derived v-ets erythroblastosis virus E26 oncogene homolog 1, which is an osteoblast differentiation related-transcriptional factor.

## Introduction

In many cell types, microvesicles (MVs) including shedding microvesicles (SMVs) and exosomes (EXOs) are released into the extracellular environment as a cell-to-cell communication tool (Bastida et al. 1984; Mack et al. 2000; Morel et al. 2004; Tesse et al. 2005; Martinez et al. 2006; Wysoczynski and Ratajczak 2009). In this study, we defined that MVs include SMVs and EXOs. These MVs contain receptor proteins, proteolytic enzymes, miRNAs, and mRNAs which are transferred into the target cell, and then affect various cell functions (Ratajczak et al. 2006; Baj-Krzyworzeka et al. 2006). In tumor cells, α-disintegrin and metalloproteinase (ADAM) and matrix metalloprotease (MMP) in MVs enhance the matrix digestion, which action facilitates the migration and metastasis of tumor cells (Gutwein et al. 2003; Mochizuki and Okada 2007). Moreover, anti-cancer drugs such as a doxorubicin decrease the levels of SMVs (Shedden et al. 2003). Thus, the MV transfer system is one of the important systems for tumor cell proliferation and progression. Osteoblastic bone metastasis in prostate cancer (PCa) patients is frequently observed as the disease progresses, and is related to high patient mortality and morbidity (Coleman 1997; Bubendoef et al. 2000; Roudier et al. 2003). In osteoblastic metastasis, a vicious cycle is established between the PCa cells and bone cells, i.e., osteoblasts and osteoclasts. PCa cells supply osteoblastic factors (e.g., bone morphogenetic proteins [BMPs], platelet-derived growth factor [PDGF], endothelin-1 [ET1]) and osteolytic factors (e.g., MMPs and vascular endothelial growth factor [VEGF]) to osteoblasts and osteoclasts, respectively, thereby allowing these cells to elaborate bone-derived growth factors (e.g., transforming growth factor-β [TGF-β], Insulin-like growth factors [IGFs]) for cell growth (Casimiro et al. 2009; Morrissey et al. 2010; Ibrahim et al. 2010). Zhang et al. (2009) showed in a recent report that heterotypic cell-to-cell contact between cells of the human prostate cancer cell line PC3 and bone marrow stromal cells (BMSC) proportionally up-regulates urokinase plasminogen activator (uPA) gene expression, which is associated with PC3 cell invasion. On the other hand, osteoblast-conditioned medium stimulates releasing of MVs from PCa cells (Festuccia et al. 1999; Millimaggi et al. 2006). Therefore, many signal exchanges are performed by direct or indirect contact between PCa cell and osteoblast during the process of bone metastasis. However, the effect of PCa-MVs on osteoblast function is not yet understood. In this study, we present evidence that PCa-MVs enhanced osteoblast differentiation mainly through the delivery of PCa-derived v-ets erythroblastosis virus E26 oncogene homolog 1 (Ets1), which is an osteoblast differentiation-related transcriptional factor.

## Materials and methods

### Reagents and materials

The 25× Complete^®^ mixture of protease inhibitors purchased from Roche (Penzberg, Germany); and Phosphatase Inhibitor Cocktail^®^ 1 and 2 from Sigma (St. Louis, MO, USA). Antibodies against human TSG101, CD9, CD81, PTHrP, Ets1, GAPDH, and mouse Ets1 were obtained from Santa Cruz Biotechnology (Santa Cruz, CA, USA). Antibody against β-actin as an internal standard was purchased from Sigma. Anti-rabbit and anti-mouse antibodies conjugated with horseradish peroxidase and the chemiluminescence (ECL) kit was obtained from GE Health Science (GE Healthcare UK Ltd., Amersham Place, Buckinghamshire, UK).

### Cell culture

PC3 and DU145 hormone-refractory human prostate cancer cells, and hormone-sensitive LNCaP cells were purchased from ATCC and cultured in RPMI 1640 medium supplemented with 10 % heat-inactivated fetal bovine serum (FBS), 100 U/ml of penicillin, and 100 μg/ml streptomycin. MVs in FBS were excluded by ultra-centrifugation (250,000×*g*, 3 h) and filtration (0.45 μm). PrEC cells were used as normal human prostate epithelial cells. Murine pre-osteoblast cell line MC3T3-E1 was obtained from RIKEN Cell Bank (Tsukuba, Ibaraki, Japan) and cultured in phenol-red free α-MEM supplemented with 10 % heat-inactivated FBS, 100 U/ml of penicillin, and 100 μg/ml streptomycin. These cells were cultured in a humidified atmosphere of 5 % CO_2_ at 37 °C.

### Electron microscopic observation

The PC3 and DU145 cells were harvested and rinsed with PBS, after which they were fixed for 30 min in 4 % paraformaldehyde and 1 % glutaraldehyde in 0.1 M phosphate buffer (pH 7.4, PB), rinsed in PB, and postfixed in 1 % osmium tetraoxide for 30 min. After having been washed with PB, the cells were progressively dehydrated by passage through a 10 % graded series of 50–100 % ethanol and then cleared in QY-1 (Nissin EM, Tokyo, Japan). They were then embedded in Epon 812 resin (TAAB Laboratories Equipment, Reading, UK); subsequently, thin sections (70 nm thickness) were cut, stained with uranyl acetate and lead citrate, and then examined by transmission electron microscopy using an Hitachi-7650 (Hitachi, Tokyo, Japan).

### Isolation of microvesicles from medium of PC3 or DU145 cell cultures

For preparation of MVs from PC3 or DU145 cells, the medium from either source was centrifuged at 1,500×*g* for 10 min to remove cells and other debris. These supernatants were then centrifuged at 250,000×*g* for 3 h at 4 °C. The centrifuged-microvesicles were resuspended in serum-free α-MEM and then filtered (0.45 μm). The filtered samples were quantified based on the protein levels by using the method of Bradford (BioRad, Hercules, CA, USA).

### Nanoparticle tracking analysis (NTA)

Microvesicles were purified from the medium of PC3 cell cultures, as described above. The microvesicle samples after passage through the 1st filter (0.22 μm) of an ExoMir kit (Bioo Scientific, Austin, TX) were used for analysis. The Nanosight LM10 nanoparticle characterization system (NanoSight, NanoSight Ltd, UK) equipped with a blue laser (638 nm) illumination was used for real-time characterization of the vesicles. The results were presented at the average value of 2 independent experiments.

### ALP staining

MC3T3-E1 cells were inoculated into 96-well plates (1 × 10^5^ cells/ml, 100 μl/well; Nunc, Roskilde, Denmark) and cultured with or without PCa-MVs (2/100 μl of MEMα/well, equivalent protein conc. 20 μg/μl) for 3 days. After incubation, the treated cells were washed twice with PBS, and then fixed with EtOH for 10 min. The ALP activity was estimated by the using a TRAP & ALP double-staining kit (Takara Bio Inc. Ohtsu, Japan) according to the manufacturer’s protocol. As a positive control, MC3T3-E1 cells were treated with 100 ng/ml of BMP-2 (R&D Systems, Minneapolis, MN, USA).

### Western blot analysis

Microvesicles was lysed with RIPA buffer containing the Complete protease inhibitor cocktail^®^ (Roche, Penzberg, Germany). Samples were then subjected to sodium dodecyl sulfate–polyacrylamide gel electrophoresis (SDS-PAGE) and electroblotted onto PVDF membranes. The membranes were incubated with a primary antibody, followed by incubation with horseradish peroxidase-conjugated secondary antibody. Immunolabeled proteins were detected by using an ECL chemiluminescence kit (GE Healthcare, Piscataway, NJ, USA) and an LAS-4000 lumino-image analyzer (Fuji film, Tokyo, Japan).

### Immunofluorescence staining

The cells were washed twice with PBS and then fixed with 4 % paraformaldehyde for 15 min at room temperature. Fixed cells were washed twice with PBS containing 10 mM glycine (PBS-G) and then treated for 5 min at room temperature with PBS containing 0.1 % Triton X-100 (Sigma) (PBS-T). Subsequently, the cells were blocked with 3 % BSA for 10 min at room temperature. After incubation, the treated cells were incubated with primary antibody (anti-human Ets1) that had been diluted with PBS-G for 1 h at room temperature. After having been washed with PBS(−) containing 0.1 % BSA, the cells were incubated with secondary antibody (Alexa Fluor-488 Rabbit IgG, Invitrogen, Carisbad, CA, USA) for 30 min at room temperature. The nuclei and cell membranes of the treated cells were further stained with Hoechst33342 (Invitrogen) and Cell Mask Orange plasma membrane stain solution (Invitrogen) for 30 min. The cells were then mounted with a drop of mounting medium (Dako cytometion fluorescent mounting medium, Dako, CA, USA) and sealed with a coverslip. Photomicrographs of mounted cells were taken with a camera-equipped fluorescence microscope (Olympus BX-50, Tokyo, Japan).

## Results and discussion

**Fig. 1:**
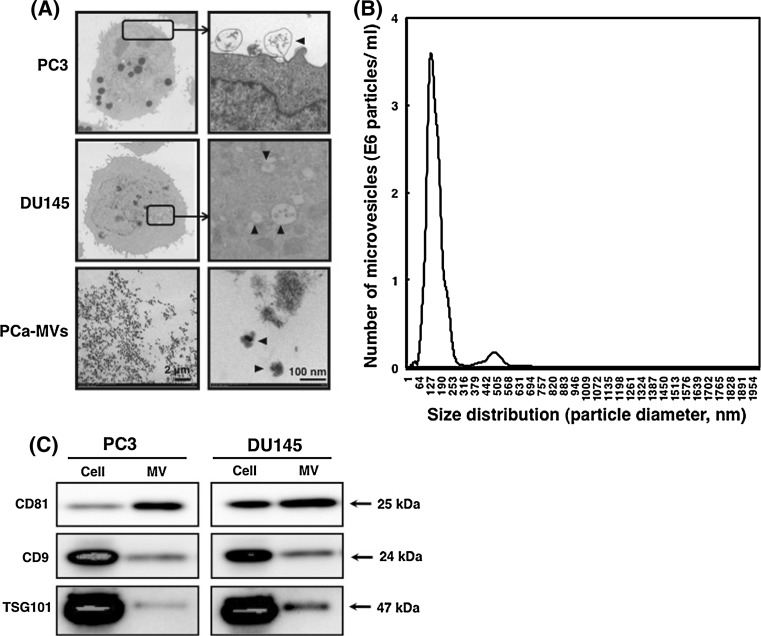
Electron-microscoipic observations of hormone-refractory-prostate cancer cell and prostate cancer cell-derived microvesicles (PCa-MVs) (**a**), analysis of microvesicle profile from human prostate cancer PC3 cells (**b**) and biochemical study of MVs from PC3 and DU145 cells (**c**). **a** Both hormone-refractory-PCa cell lines PC3 cells and DU145 cells shed microvesicles from their cell membrane. The *black arrowheads* indicate PCa-MVs. The *right* photos of PC3 and DU145 cells show enlarged views of *rectangular* areas in the *left* photos. **b** MVs were isolated from PC3 cells and measured with NTA. For preparation of MVs from PC3 cells, the medium from either source was centrifuged at 1,500×*g* for 10 min to remove cells and other debris. These supernatants were then centrifuged at 250,000×*g* for 3 h at 4 °C. The centrifuged-microvesicles were resuspended in serum-free α-MEM and then filtered (0.45 μm). The microvesicle samples after passage through the 1st filter (0.22 μm) of an ExoMir kit were used for analysis. The Nanosight LM10 nanoparticle characterization system (NanoSight, NanoSight Ltd, UK) equipped with a blue laser (638 nm) illumination was used for real-time characterization of the vesicles. The results were presented at the average value of 2 independent experiments. The number of microvesicles (E6 particles/ml) and the size distribution (particle diameter, nm) are shown on the *y* axis and *x* axis, respectively. **c** Western blots of MV-related proteins, i.e., TSG101, CD9, and CD81. MVs were prepared from the culture medium by ultracentrifugation (250,000×*g* for 3 h, 4 °C). The microvesicles were further filtered (0.45 μm) and then resuspended in RIPA buffer containing the complete protease inhibitor cocktail. Three micrograms protein per lane was loaded. The relative expression levels of the MV-related proteins indicate a specificity for MVs

**Fig. 2:**
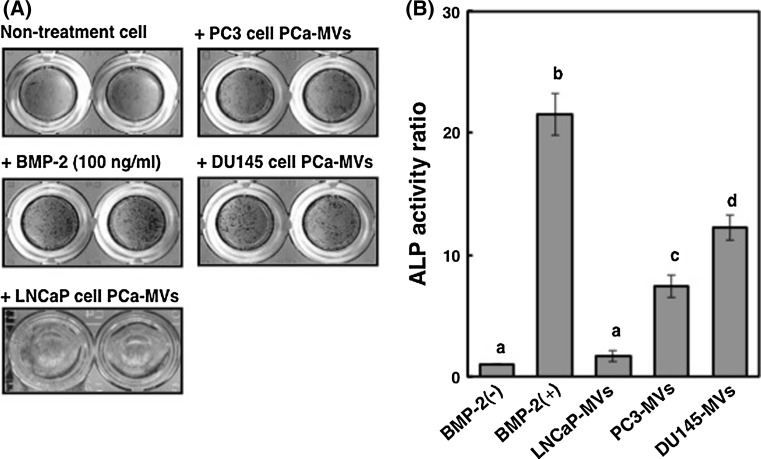
Effect of the PCa-MVs on differentiation of murine preosteoblast MC3T3-E1 cells. MC3T3-E1 cells were inoculated into 96-well plates (1 × 10^5^ cells/ml, 100 μl/well) and cultured with or without PCa-MVs (2/100 μl of MEMα/well, equivalent protein conc. 20 μg/μl) for 72 h. The ALP activity was estimated by using a TRAP & ALP double-staining kit (**a**). As a positive control, MC3T3-E1 cells were treated with 100 ng/ml of BMP-2. In order to quantify the levels of ALP activity based on the positively stained area on the each plate well field was analyzed using Image J software, a public domain image processing and analysis program developed by the NIH (**b**). The ALP activity was shown as a ratio to the BMP-2 (−) control

As shown in Fig. 1a, the cells of hormone-refractory PCa cell lines PC3 and DU145 cells in logarithmic growth phase shed MVs from their plasma membrane (Fig. 1a; upper panel and middle panel, respectively). The diameters of these MVs were approximately 50–100 nm (Fig. 1a, lower panel). NTA (nanoparticle tracking analysis) indicated that the microvesicles from PC3 cells were 139 nm in diameter, as shown by the peak in the size-distribution graph (Fig. 1b). The biochemical characterization of MVs indicated a difference in expression levels of MV-related TSG101, tetraspanin CD9 and CD81 between the cells and MVs (Fig. 1c). CD81 was relatively specific for MV among them. Thus, we confirmed that the PC3 and DU145 cells released MVs into their culture medium. To examine the effect of PCa-MVs on osteoblast differentiation, we added PCa-MVs in suspension to murine pre-osteoblast MC3T3-E1 cell cultures and then incubated the cells for 72 h at 37 °C. Thereafter, the induction of differentiation was estimated by ALP staining. The number of the MVs incubated in a well was approximately 1 × 10^7^ particles. PCa-MVs prepared from either PC3 or DU145 cell cultures significantly facilitated osteoblast differentiation, but the PCa-MVs from LNCaP cells did not (Fig. 2a, b). The differentiating activity of these PCa-MVs was in the order of BMP-2 (100 ng/ml, positive control) > DU145 > PC3.

**Fig. 3:**
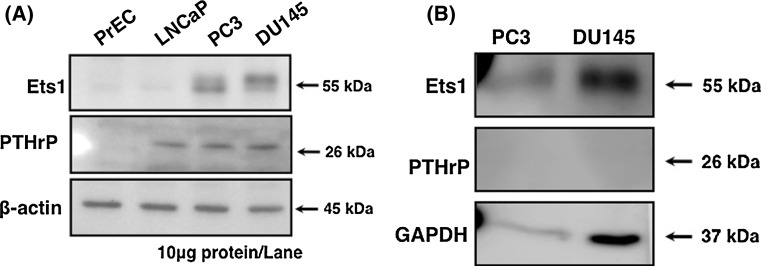
Protein expression levels of Ets1 and PTHrP in each human PCa cell line (**a**) and determination of Ets1 and PTHrP in MVs from both hormone-refractory PCa cells (**b**). **a** MVs were prepared from the culture medium by ultracentrifugation and filtration, as described in Fig. 2b. Lysates of the MVs were prepared and subjected to western blot analysis for Ets1, PTHrP, and GAPDH (PC3 cells, 10 μg protein/lane; DU145 cells, 50 μg protein/lane). **b** Lysates of the indicated cell types were prepared and subjected to western blot analysis for Ets1, PTHrP, and β-actin (10 μg protein/lane). Antibodies against β-actin was used as an internal standard. PrEC cells were used as an example of normal human prostate epithelial cells

To disclose the mechanisms underlying the stimulation of osteoblast differentiation by PCa-MVs, we further focused on the protein levels of Ets1 and parathyroid hormone-related protein (PTHrP) in PCa cells and PCa-MVs. Tumor-derived PTHrP up-regulates bone remodeling, leading to the release of numerous bone-derived growth factors from osteoblasts or osteoclasts for PCa cell growth (Casimiro et al. 2009; Morrissey et al. 2010; Ibrahim et al. 2010). Ets1 is a proto-oncogene protein that is highly expressed tumor cells and regulates the expression of MMP-1, -3, -9, and uPA (Sementchenko and Watson 2000). In osteogenesis, Ets1 also regulates the expression of several proteins such as osteopontin (OPN), tenascin-C, and procollagen (Sato et al. 1998; Raouf and Seth 2000). Moreover, Ets1 activates PTHrP gene expression via the binding of *PTHrP* P3 promoter region with cAMP response element binding protein (CREB) (Hamzaoui et al. 2007). Thus, PTHrP and Ets1 are important molecules in tumor metastasis. The data presented in Fig. 3a show that both hormone-refractory PCa cell lines, PC3 and DU145, highly expressed Ets1 and PTHrP but that neither protein was detected in PrEC normal human prostate epithelial cells. Interestingly, LNCaP human hormone-sensitive PCa cells did not express Ets1 but did express PTHrP. On the other hand, Ets1 was detected in MVs from both hormone-refractory PCa cell lines: but PTHrP was detected in neither of them (Fig. 3b). From these results, we propose that the bone-derived growth factors such as BMPs, Ets1, and PTHrP were wrapped in PCa-MVs in accordance with some certain rules rather than enclosed randomly. Ets1 acts as a transcription factor in osteoblasts during their differentiation. Therefore, the PCa-MVs delivery system, which affords fusion of MVs to the plasma membrane of target cells, is a reasonable transfer model for Ets1. In contrast to Ets1, PTHrP binds to the PTH receptor (PTHR) expressed on the surface of osteoblasts, which cells produce receptor activator of nuclear factor kappa-B ligand (RANKL) and monocyte chemoattractant protein-1 (MCP-1) (Lu et al. 2007; Liao et al. 2008). Hence, PTHrP needs to interact with PTHR for osteoblast differentiation. Thus, PC3 and DU145 cells highly expressed Ets1 and PTHrP, but in their MVs PTHrP was hardly detected by western blot analysis (Fig. 3). Also, LNCaP cells expressed PTHrP but not Ets1, the MVs from LNCaP cells were not able to induce the differentiation (Fig. 2). These findings indicate that the higher expressed Ets1 is a possible candidate inducer of osteoblast differentiation.

**Fig. 4:**
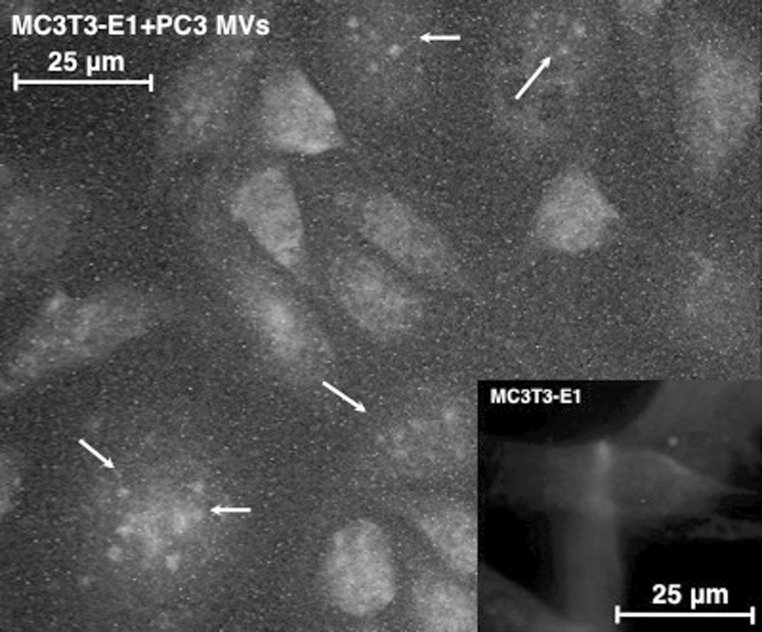
Immunofluorescence analysis of PCa-MV-treated MC3T3-E1 cells. MC3T3-E1 cells were incubated with MVs from PC3 cells. After incubation, the fixed MC3T3-E1 cells were incubated with the primary antibody (anti-human Ets1) for 1 h at room temperature. After having been washed with PBS(−) containing 0.1 % BSA, the cells were incubated with secondary antibody (*Green* Alexa Fluor-488 Rabbit IgG) for 30 min at room temperature. The nuclei and cell membranes of the treated cells were further stained with Hoechst33342 (*Blue*) and Cell Mask Orange plasma membrane staining solution (*Red*) for 30 min. Photomicrographs of mounted cells were taken with a fluorescence microscope. The *white arrows* indicate the Ets1 discharged from PCa-MVs. (Color figure online)

In order to investigate intracellular location of the Ets1 delivered to the osteoblast via PCa-MVs, we used immunofluorescence staining. Although much of the Ets1 from the PCa-MVs adhered to the osteoblast cell surface, a part of it was transferred into the nuclei (Fig. 4). However, we did not measure the expression levels of Ets1-regulated molecules such as *PTHrP*, *OPN*, *tenascin*-*C*, and *procollagen*. Previously, we reported that miR-208 targets Ets1, which leads to attenuation of the differentiation with downregulation of OPN and Runx2 (Itoh et al. 2010). So, the question still remains as to whether or not the Ets1 transferred via PCa-MVs acts in nuclei as a transcription factor. We cannot deny the possibility that not only Ets1 protein but also other proteins, mRNAs and miRNAs related to osteoblast differentiation may be transferred into the cells via the PCa-MVs. Further study is required to answer these questions.

In summary, Ets1-containing MVs from hormone-refractory PCa cells were transferred into osteoblasts, and the Ets1 discharged into the cytoplasm functioned to induce differentiation. Our findings thus suggest that PCa-MVs acted as a cell-to-cell communication tool in osteoblastic metastasis.
